# Partial Segmental Thrombosis of the Corpus Cavernosum Associated With Inappropriate Sildenafil Use

**DOI:** 10.7759/cureus.53462

**Published:** 2024-02-02

**Authors:** Henry G Danchi, Carl Kay, Steven R Hole, Austin Baraki

**Affiliations:** 1 Internal Medicine, Brooke Army Medical Center, San Antonio, USA; 2 Gastroenterology, Brooke Army Medical Center, San Antonio, USA; 3 Radiology, Brooke Army Medical Center, San Antonio, USA; 4 Internal Medicine, William Beaumont Army Medical Center, El Paso, USA

**Keywords:** sexual intercourse, rivaroxaban, sildenafil, partial priapism, pstcc, partial segmental thrombosis of the corpus cavernosum

## Abstract

The case presented is of a 39-year-old male with severe right groin pain and perineal pain the morning after sexual intercourse with the use of sildenafil without a diagnosis of erectile dysfunction. Partial segmental thrombosis of the corpus cavernosum (PSTCC) was diagnosed using magnetic resonance imaging and treated with direct oral anticoagulation without complications. Sildenafil use has been noted as an inciting factor for PSTCC in only two other cases of less than 60 cases reported in the literature and has even been used successfully as a component of therapeutic management of PSTCC in another previous case.

## Introduction

PSTCC is a rare urologic condition that generally affects young men. It was first described in 1976 by Grossman et al. and described as “partial priapism.” Management has been evolving, as demonstrated by the wide variety of modalities spanning from medical management to surgical intervention [1]. Treatments have included conservative analgesia and supportive care, anticoagulation, nonspecific phosphodiesterase inhibitors, shunt procedures, and surgical evacuation [2]. In addition to the aforementioned therapies, management hinges on determining a precipitant. PSTCC is associated with hypercoagulable disorders, illicit drug use, prior history of priapism, tamsulosin, prolonged air travel, mechanical trauma from bicycle riding, and vigorous sexual activity; however, in most cases, no cause is identified [3]. We present a case of PSTCC associated with inappropriate use of sildenafil, which was successfully treated with oral systemic anticoagulation after stopping the presumed offending agent.

## Case presentation

**Figure 1:**
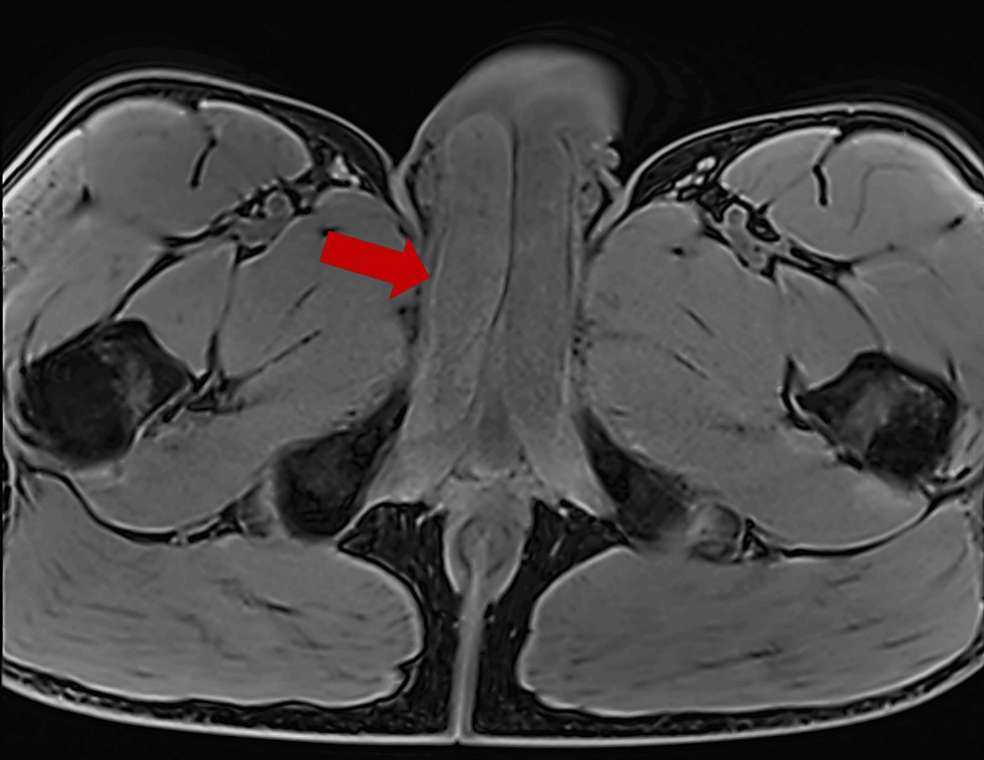
Transverse fat-saturated T1-weighted MRI. Enlarged appearance of the proximal portion of the right corpus cavernosum compared to the left with no definite T1 signal abnormality. A red arrow points at the right corpus cavernosum.

**Figure 2:**
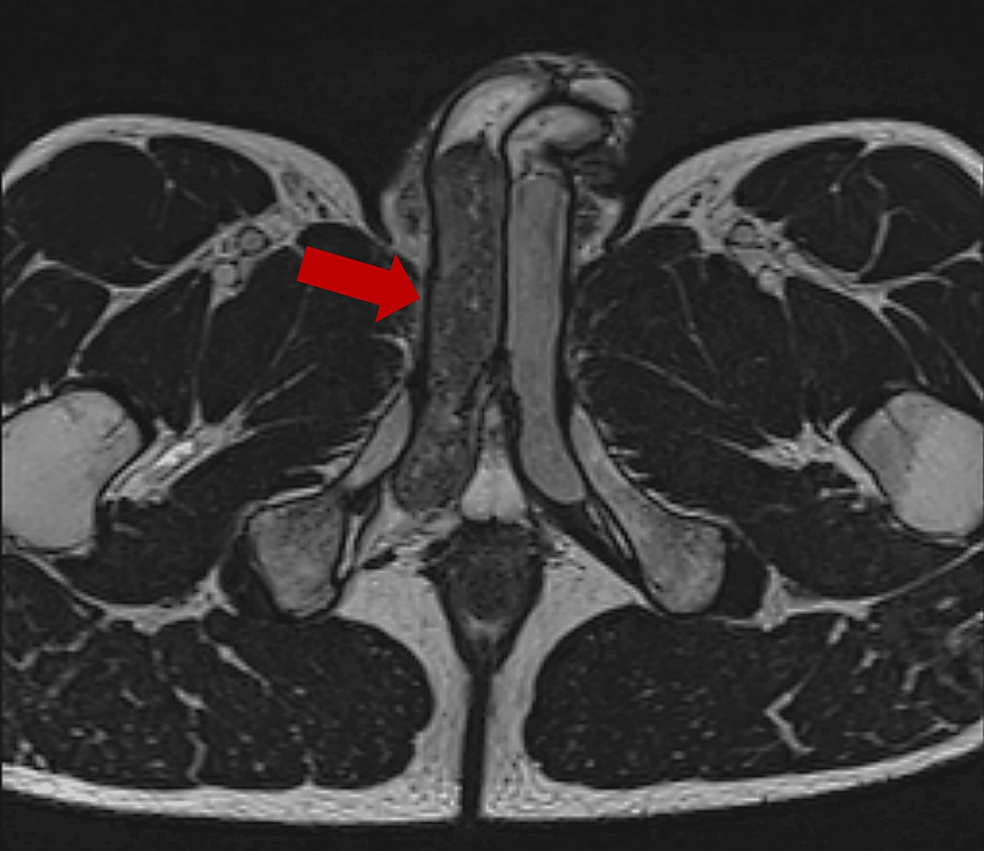
Transverse T2-weighted MRI. Enlarged appearance of the proximal portion of the right corpus cavernosum compared to the left with hypointense T2 signal intensity. A red arrow point at the right corpus cavernosum.

A 39-year-old male with no significant past medical history presented with severe right groin and perineal pain for three days. He reported having normal sexual intercourse before the onset of symptoms. He had been intermittently using sildenafil 50 mg, approximately two to three times weekly, before sexual activity without a medical diagnosis of erectile dysfunction. He denied a history of priapism, mechanical trauma, vigorous sexual activity, drug use, or prolonged air travel. His exam was notable for an enlarged right proximal corpora but no evidence of priapism. Initial ultrasound imaging revealed mild asymmetric fullness of the right corpus spongiosis compared to the left. There was no evidence of surrounding edema, suspicious soft tissue mass, hypervascularity, or fluid collection. Follow-up contrasted magnetic resonance imaging revealed a diffusely enlarged appearance of the proximal portion of the right corpus cavernosum compared to the left, with hypointense T2 signal intensity and no definite T1 signal abnormality (Figures 1, 2). Overall, imaging findings were most consistent with partial segmental thrombosis of the right proximal corpus cavernosum.

Urologic evaluation included aspiration of corporal fluid, which revealed unremarkable cytology without evidence of infiltrative malignancy. The lab evaluation was negative for sickle cell or findings on peripheral blood smears. Upon discharge, he was advised to discontinue the use of sildenafil. He was managed with conservative analgesia and started on direct oral anticoagulation with rivaroxaban 15mg twice daily for three weeks, followed by 20mg once daily for six months. The pain associated with the thrombus resolved completely over the following two to three weeks. He had no bleeding complications. He reported no recurrence, erectile dysfunction, or impotence after completion of the course of system anticoagulation with rivaroxaban.

## Discussion

PSTCC is a rare condition that usually occurs in young men, with less than 60 cases reported in the literature [4]. While a cause or precipitant is often difficult to elucidate, the patient’s minimal past medical history and unremarkable initial laboratory work-up make it possible to identify possible risk factors, including inappropriate sildenafil use. Given the temporal relationship to sildenafil use without a diagnosis of erectile dysfunction and the absence of other obvious precipitants, we believe his inappropriate sildenafil use contributed to his abnormal thrombus formation. Unfortunately, the patient declined further evaluation on follow-up, including studies for hypercoagulable disorders. Therefore, this cannot be ruled out as a possible contributor to the event.

To the best of our knowledge, there have only been two other case reports of PSTCC associated with sildenafil use [5,6]. Paradoxically, phosphodiesterase-5 (PDE-5) inhibitors have even been used as part of a therapeutic regimen in conjunction with oral anticoagulation [4]. Our hypothesis of how sildenafil may predispose males towards developing PSTCC may be consistent with the “two-hit model” first suggested by Ilicki et al. [7]. The first hit is the presence of a transverse membrane separating the proximal root from the distal body of the corpus cavernosum. The fibrous, semi-permeable membrane may occur congenitally or post-traumatically. The second hit, such as trauma, medications, hypercoagulable state, or a combination thereof, results in the formation of clots along the membrane within the proximal corpus. Venous congestion and micro-trauma from perineal compression likely contribute to clotting. This occurs through activities such as bicycle riding and sexual intercourse. Similarly, sildenafil use may result in increased venous stasis from prolonged engorgement. Combined with micro-trauma from sexual activity, this appears to have resulted in the development of PSTCC in this patient. 

Moreover, to the best of our knowledge, this is the second case of PSTCC managed with direct oral anticoagulation; previously proposed systemic anticoagulation regimens were limited to low-molecular-weight heparin (1 mg/kg dosing), heparin, or acetylsalicylic acid. This simplified regimen allows for better medication compliance. Our case serves as further evidence for future management with oral anticoagulation.

## Conclusions

This case represents the third-ever reported case of PSTCC associated with sildenafil use, as well as the second case treated with direct oral anticoagulation. No other causes or risk factors were found outside of sexual intercourse and sildenafil use that preceded symptom onset. Though a complete evaluation for hypercoagulable disorders was not able to be completed, the two-hit model would suggest that the patient may have been predisposed to the condition by the presence of a transverse membrane of the corpus cavernosum, which then allowed for thrombus formation under the conditions of micro-trauma and venous stasis secondary to sexual intercourse and sildenafil use, respectively. Management with direct oral anticoagulation resulted in uncomplicated clinical improvement and may serve as further evidence for future management of PSTCC as an alternative to heparin and low-molecular-weight heparin.
